# Evaluating the Effectiveness of Radiofrequency in Multimodal Physiotherapy for Postpartum Pelvic Pain: The RASDOP Protocol—A Mixed-Methods Study †

**DOI:** 10.3390/jcm14051489

**Published:** 2025-02-23

**Authors:** Beatriz Navarro-Brazález, Laura Lorenzo-Gallego, Paula Rangel-de la Mata, María Torres-Lacomba, Fernando Vergara-Pérez, Beatriz Sánchez-Sánchez, Nuria Izquierdo-Méndez

**Affiliations:** 1 Physiotherapy in Women’s Health (FPSM) Research Group, Department of Nursing and Physical Therapy, Faculty of Medicine and Health Sciences, Universidad de Alcalá, 28805 Alcalá de Henares, Madrid, Spain; b.navarro@uah.es (B.N.-B.); laura.lorenzo@edu.uah.es (L.L.-G.); paula.rangel@uah.es (P.R.-d.l.M.); fernando.vergara@uah.es (F.V.-P.); beatriz.sanchez@uah.es (B.S.-S.); 2 Ramón y Cajal Institute of Health Research–IRYCIS, Hospital Universitario Ramón y Cajal, 28034 Madrid, Spain; 3 Gynecology and Obstetrics Service of the Príncipe de Asturias University Hospital, 28805 Alcalá de Henares, Madrid, Spain; nizquierdo@salud.madrid.org

**Keywords:** dyspareunia, myofascial pain syndrome, pelvic floor muscles, pelvic pain, physiotherapy, postpartum, radiofrequency

## Abstract

**Background/Objectives**: Approximately 30% of women experience pelvic pain one year after vaginal delivery, and this increases to 50% during vaginal intercourse. Multimodal physiotherapy is the first-line treatment for myofascial pain of the pelvic floor muscles (PFM), often incorporating emerging technologies like radiofrequency, despite limited evidence supporting its use. The RASDOP study aims to (i) evaluate the effectiveness of multimodal physiotherapy combining therapeutic education, PFM training, and myofascial pain syndrome (MPS) management with or without radiofrequency, in reducing pain and improving sexual function postpartum; and (ii) explore the barriers and facilitators influencing adherence and perceived self-efficacy towards physiotherapy treatment in women with postpartum pelvic pain. **Methods**: This study employs a randomized clinical trial with a blinded examiner and two parallel groups, followed by a qualitative phenomenological study. A total of 124 postpartum women with pelvic pain (≥4 cm on the visual analogue scale) will be randomized into two groups: a multimodal physiotherapy group (therapeutic education, MPS treatment, and PFM training) and a radiofrequency + multimodal physiotherapy group (same treatment with additional non-ablative radiofrequency). Both groups will receive 12 supervised individual sessions. Assessments will be conducted pre- and post-intervention, and at 3-, 6-, and 12-month stages post-treatment. Primary outcomes include pain intensity and sexual function, while secondary outcomes involve PFM tone, strength, MPS presence, levator ani length, and distress caused by pelvic floor dysfunction. The qualitative study will utilize semi-structured interviews and focus groups analyzed thematically. **Results**: Radiofrequency is expected to enhance pain relief, sexual function, and PFM relaxation. Insights into adherence and barriers will aid in personalizing physiotherapy interventions. **Conclusions**: The RASDOP study will provide evidence on the safety and efficacy of radiofrequency in postpartum pelvic pain treatment and shed light on women’s experiences to improve therapeutic outcomes.

## 1. Introduction

International scientific and professional societies describe pelvic pain as a significant health concern, particularly when it persists over time [1,2,3], and it is associated with impaired sexual function [4]. Both sexes can experience pelvic pain-related sexual dysfunction, though its prevalence appears to be higher in women. The prevalence of non-cyclic chronic pelvic pain in women is estimated to reach up to 30% [5], while approximately 40–50% of women, regardless of age, have experienced sexual problems at least once in their lifetime [6]. The association between pelvic pain, urogenital pain, and vulvovaginal pain in the female population is strongly linked to alterations in sexual activity, leading to the use of terms that relate pain to sexual dysfunction, such as vaginismus and dyspareunia [2,3,7]. However, this pain can also manifest during other daily activities, be accompanied by other pelvic floor dysfunctions [8], or affect different phases of the sexual response cycle, such as desire, arousal, or orgasm [9].

The etiology and risk factors associated with pain localized to the pelvic areas are multifactorial [10,11]. Pregnancy and vaginal delivery, whether spontaneous or instrumental, have been identified as predisposing and perpetuating risk factors of pelvic and genital pain [12]. This positive association between pregnancy, childbirth, and pelvic and genital pain is observed in up to 34% of women one year postpartum, increasing to 33–52% when pain is triggered during vaginal intercourse, a condition referred to as dyspareunia [12,13]. Furthermore, when considering other domains involved in sexual function, such as arousal, desire, lubrication, or orgasm, a cross-sectional study conducted in 325 women reported that over 60% experienced sexual dysfunction during the first year postpartum, and 70% reported sexual dissatisfaction [14]. The hormonal and biomechanical adaptations of the pelvis to accommodate the baby during pregnancy, the overstretching of the pelvic floor muscles (PFM) and their neural structures during childbirth [15], as well as the lacerations or trauma that may occur in the perineum during the expulsive phase [16,17], appear to be significant sources of pain. Additionally, increased tenderness of the PFM has been observed in women with chronic pelvic pain compared to those without pain, and this has also been associated with dyspareunia [18]. The presence of myofascial pain syndrome (MPS) in the PFM and other muscles related to the abdominopelvic cavity may result in pain and sexual dysfunction, as well as being associated with other symptoms affecting the urogenital and gastrointestinal systems, such as difficulty emptying the bladder, a sensation of vaginal heaviness, or pain during defecation, among others [19].

When addressing pelvic pain, especially associated with tenderness, high tone or the presence of MPS in PFM, multimodal physiotherapy is the first line treatment [2,20]. The multimodal physiotherapy includes different strategies such as PFM training (PFMT), biofeedback, dilators, electrical stimulation, manual therapy or education [21]. Specific PFMT has been shown to be effective in reducing pain in women with provoked vestibulodynia [22] and vaginismus [23], improvements that appear to increase when combined with local manual therapy, resulting in a reduction in pain intensity and an improvement in sexual function [24]. Patient therapeutic education for pain has been proven to be an effective tool that provides self-management strategies for patients [23,25,26] addressing misconceptions, beliefs, and cognitive and behavioral aspects that can modify pain intensity, even preventing its perpetuation or worsening. Furthermore, in clinical practice, non-ablative radiofrequency is increasingly being incorporated into the management of pelvic pain. This tool has made a significant impact in the field of multimodal physiotherapy, and it seems to induce local biological changes by stimulating fibroblasts, leading to the production and reorganization of collagen and elastin fibers [27]. This could, for example, improve the healing of an episiotomy and secondarily enhance the muscular qualities of the PFM. In women with chronic pelvic pain and dyspareunia, radiofrequency appears to reduce pain, improve sexual function, enhance PFM strength, and alleviate urinary incontinence symptoms. However, the quality of the evidence, the heterogeneity of the treatment protocols, and the small sample sizes highlight the need for further studies to confirm its effectiveness [28]. Only one randomized clinical trial (RCT) conducted in postpartum women has analyzed the efficacy of radiofrequency on pelvic pain 3 to 6 months after delivery. This study compared five sessions of radiofrequency with five sessions of manual therapy targeting the PFM. Both groups reported improvements in subjective perceptions of pain relief and overall improvement; however, only the radiofrequency group demonstrated increased vaginal laxity [29]. Nevertheless, no significant improvements in PFM neuromuscular activity were observed, likely due to the absence of a multimodal physiotherapy approach that included PFMT and educational strategies. Additionally, the study did not evaluate sexual function and focused solely on short-term effects, despite this population being at risk of developing new pelvic floor symptoms, including pelvic pain, when resuming to work, sports, or sexual activity. Therefore, it is essential to incorporate preventive therapeutic strategies and home self-management techniques during the postpartum period [30].

The long-term maintenance of positive outcomes depends not only on the treatment but also on behavioral changes and adherence to the physiotherapist’s recommendations [31]. Therefore, exploring and understanding women’s motivation, perceived self-efficacy, and expectations, among other factors, seems essential to ensuring the long-term effectiveness of conservative treatments [32].

The RASDOP study is designed to (i) compare standard multimodal physiotherapy for postpartum pelvic pain with an experimental approach that includes radiofrequency therapy, whose efficacy and safety remain unknown; and (ii) explore factors influencing therapeutic adherence and perceived self-efficacy, identifying the challenges and facilitators that affect postpartum women in adopting and maintaining recommended practices for long-term benefits.

Our hypothesis is that the inclusion of radiofrequency within a multimodal physiotherapy treatment enhances its efficacy in managing postpartum pelvic pain by reducing pain, improving sexual function, eliminating the presence of MPS in the PFM, and improving PFM qualities. Additionally, factors influencing postpartum women’s adherence or non-adherence to a conservative treatment plan for pelvic pain will be explored. These findings will assist clinicians in decision-making regarding the inclusion of technologies such as radiofrequency in the management of pain in this population and provide insights to guide the development of a biopsychosocial approach to postpartum pain treatment.

## 2. Materials and Methods

### 2.1. Study Design

The RASDOP study is a mixed-methods study comprising a single-blind RCT and a qualitative component. The RCT will follow a two-parallel-arm design. Outcome assessment will be blinded, meaning that the evaluators conducting the assessments will not be aware of the participants’ group allocation to reduce detection bias. However, due to the nature of the interventions, neither participants nor treating physiotherapists will be blinded. In parallel, a qualitative study will be conducted using a phenomenological approach, involving individual and group interviews with women from the RCT.

This study has been approved by the Research and Animal Experimentation Ethics Committee of the University of Alcalá under registration number CEIP/2023/3/056. This study has also been registered on the ClinicalTrials.gov platform under the identification code NCT06469632.

### 2.2. Randomized Controlled Clinical Trial

All procedures will be conducted in compliance with the ethical principles of the Declaration of Helsinki and guided by the CONSORT recommendations for clinical trials [33]. The presentation of this protocol follows the guidelines of the SPIRIT 2013 Statement for protocol publication [34].

#### 2.2.1. Study Participants

Postpartum women experiencing pelvic pain following a vaginal delivery, referred by the Gynecology and Obstetrics Department of Príncipe de Asturias University Hospital for physiotherapy assessment. Physiotherapy assessments and treatments will be conducted at the laboratory of the Physiotherapy in Women’s Health (FPSM) Research Group at the University of Alcalá.

**Figure 1:**
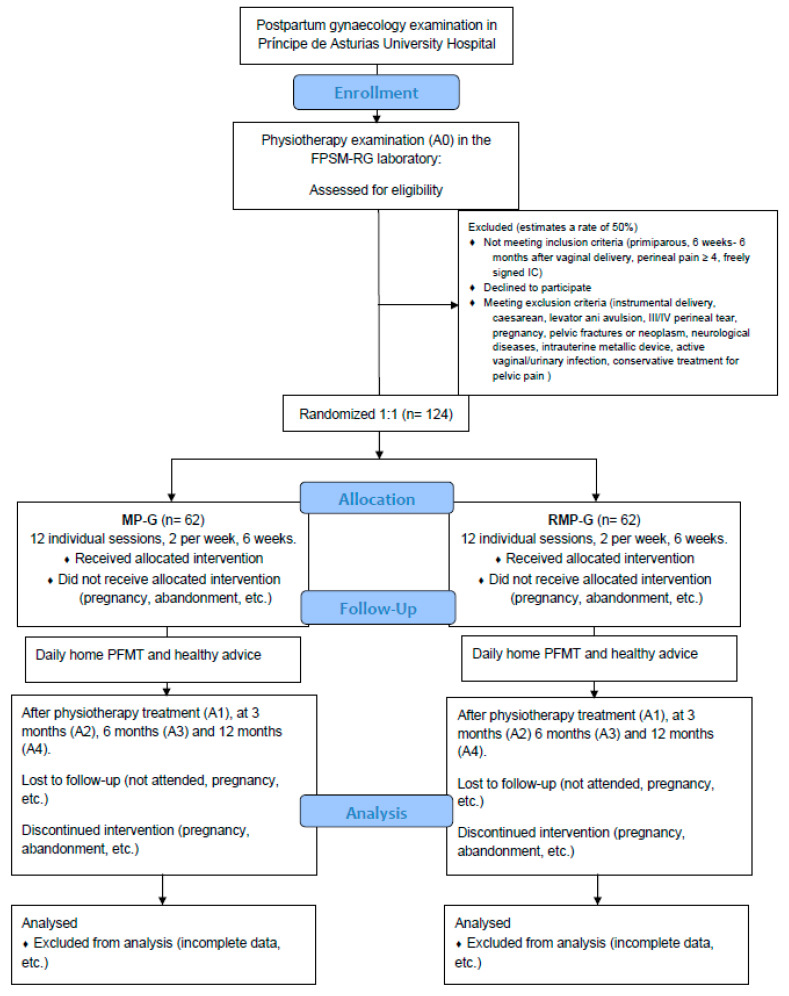
CONSORT follow Diagram. FPSM-RG: Physiotherapy in Women’s Health Research Group, A: assessment; IC: informed consent; MP-G: multimodal physiotherapy group; RMP-G: radiofrequency + multimodal physiotherapy group; PFMT: pelvic floor muscle training.

The inclusion criteria will be primiparous women who have had a vaginal delivery, within the past 6 weeks–6 months, experiencing postpartum pelvic pain with an intensity of ≥4 cm on the visual analog scale, and who have read, understood, and freely signed the informed consent. Women will be excluded if they are multiparous, have undergone instrumental vaginal delivery or cesarean section, experienced avulsion of the levator ani muscle, sustained a third- or fourth-degree perineal tear, have a history of pelvic fractures and/or neoplasms, are pregnant, have neurological conditions, an active vaginal or urinary tract infection, an intrauterine device with metallic components, or cognitive, auditory, and/or visual impairments that prevent them from understanding the information, responding to questionnaires, providing consent, and/or participating in the study. Women who have received or are currently undergoing medical or physiotherapeutic treatment for pelvic pain will also be excluded. The enrollment, allocation, treatment, and follow-up scheme for the participants is shown in Figure 1.

#### 2.2.2. Sample Size

The sample size was calculated based on the results obtained by Citak et al. [35], using the Female Sexual Function Index (FSFI) scores as a reference and the statistical program GRANMO 8.0 (Datarus, Barcelona, Spain). Assuming a two-tailed test with an alpha risk of 0.05 and a statistical power greater than 0.8, 62 participants per group will be required to detect a difference of 2.4 points or greater. A common standard deviation of 4.5 was assumed. Additionally, a 10% dropout rate was accounted for in the estimation.

#### 2.2.3. Physiotherapy Assessments

Physiotherapy assessments will be conducted by a physiotherapist specializing in Pelvic and Women’s Health (PT1) with over 10 years of experience, who will be blinded to the participants’ intervention group. The physiotherapy assessment will include an interview to collect socio-demographic and clinical history data, as well as a physical examination (Table 1). The outcome variables of the study that will be collected at all assessments are as follows:

- Primary outcomes:
  (1) Pelvic pain: The perceived pain intensity will be measured using the Visual Analog Scale (VAS). This scale consists of a 100 mm horizontal line, where the participant marks the intensity of their pain, with the left margin of the line representing “no pain” and the right margin representing “the worst possible pain.” A minimum detectable change (MDC) of 15 mm is required for clinical significance [36].
  (2) Female sexual function: The Spanish version of the FSFI will be used [37]. The FSFI questionnaire has a scoring range from 2 to 36 points, with higher scores indicating better sexual function. It assesses six domains: desire, arousal, lubrication, orgasm, satisfaction, and pain. A change of 6 points will be considered clinically relevant [38].
- Secondary outcomes:
  (1) Myofascial pain syndrome (MPS): The presence of MPS will be identified by assessing myofascial trigger points (MTrPs) in the abdominal muscles and PFM. To diagnose MPS, at least one active MTrP must be identified. The essential clinical criteria proposed by Travell and Simons will be followed (Table 2) [39]. Additionally, confirmatory observations will be recorded, such as the presence of referred pain elicited by palpating the MTrP [40].
  (2) Pelvic floor dysfunction perceived distress: Evaluated using the transculturally validated Spanish version of the Pelvic Floor Distress Inventory–short form (PFDI-20) [41]. The PFDI-20 is scored from 0 to 300, with higher scores indicating greater distress caused by pelvic floor dysfunctions. It assesses lower urinary tract symptoms, anorectal symptoms, and pelvic organ prolapse symptoms. A minimal important change of 23% will be considered clinically significant [42].
  (3) Impact of pelvic floor dysfunctions: Assessed using the Spanish version of the Pelvic Floor Impact Questionnaire–short form (PFIQ-7) [41], which includes three subscales related to urinary, intestinal, and prolapse symptoms. Each subscale is scored from 0 to 100, where a higher score indicates a greater impact and worse quality of life. An MDC of 16% is required for clinical significance [43].
  (4) Pelvic floor muscles tone: PFM tone will be assessed using vaginal dynamometry (Pelvimetre™, Phenix Vivaltis, Montpellier, France). The participant will be in the lithotomy position, and the lowest value automatically recorded by the device will be taken after instructing the woman to relax her PFM. A MDC of 5 g will be considered [44].
  (5) Pelvic floor muscle strength: PFM strength will be measured in the lithotomy position by requesting three maximal PFM contractions. The average value of the three contractions will be considered. A vaginal dynamometer (Pelvimetre™, Phenix Vivaltis, Montpellier, France) will be used for the measurement, and a MDC of 50 g will be considered [44].
  (6) Levator hiatus length: Using 2D transperineal ultrasound (Mindray M7, Shenzhen, China) in the sagittal plane, the distance between the inferior margin of the pubic symphysis and the anorectal angle will be assessed [45]. Measurements will be taken at rest, during maximal PFM contraction, and during a strain effort.
  (7) Perceived self-efficacy: assessed using the Broome Pelvic Muscle Self-Efficacy Scale–Spanish version [46]. This scale evaluates expectations of self-efficacy in performing specific PFM exercises and expectations regarding the anticipated outcomes. The maximum score is 100 points, indicating high self-efficacy, while a score below 33 points indicates low self-efficacy [46].

**Table 1:** Study assessed variables and evaluations.

Assessed Variables	Assessment				
	A0	A1	A2	A3	A4
Sociodemographic variables (address, telephone number, mail, profession, studies, date of birth)	X	only if changes	only if changes	only if changes	only if changes
General clinical data (weight, height, medication, diagnosed pathologies, surgeries, allergies, sports routines, healthy habits)	X	only if changes	only if changes	only if changes	only if changes
Gynecological history (pregnancies, weight gain during pregnancy, type of delivery, presence and degree of tear, presence of episiotomy, weight and length of the baby, breastfeeding, menstruation, symptoms of pelvic floor dysfunction)	X	only if changes	only if changes	only if changes	only if changes
Pelvic floor observation (contraction capacity, relaxation capacity, apnea, parasitic muscle contraction, reflexes, symmetries, perineal scars)	X	X	X	X	X
PFM objective evaluation (Modified Oxford scale, tone and strength by vaginal dynamometry; rest, PFM contraction and bearing down by transperineal ultrasound)	X	X	X	X	X
Diagnosis of MPS (pain: description, intensity, body chart, triggering factor; palpation of abdominal and pelvic floor surface and deep muscles)	X	X	X	X	X
Questionnaires (FSFI, PFDI-20, PFIQ-7, Broome scale)	X	X	X	X	X

A0: pre-treatment assessment, A1: post-treatment assessment, A2: 3-month assessment after treatment, A3: 6-month assessment after treatment, A4: 12-month assessment after treatment, PFM: pelvic floor muscles, MPS: myofascial pain syndrome, FSFI: Female Sexual Function Index, PFDI-20: Pelvic Floor Distress Inventory–short form, PFIQ-7: Pelvic Floor Impact Questionnaire–short form.

**Table 2:** Diagnostic clinical criteria for identifying active and latent myofascial trigger points.

Essential Criteria	Active MTrPs	Latent MTrPs
Palpable taut band	X	X
Exquisite local tenderness upon pressure over a nodule within the taut band	X	X
Patient recognition of their typical pain upon pressure applied to the tender nodule	X	
Painful limitation of the range of motion upon full muscle stretch	X	X
**Confirmatory Observation**		
Visual or tactile identification of a local twitch response	X	X
Pain or altered sensitivity (in the predictable distribution of an MTrP in that muscle) upon compression of the tender nodule	X	X

MTrPs: myofascial trigger points.

The Spanish versions of FSFI, PFDI-20, and PFIQ-7 questionnaires, and the Broome scale are self-administered. Participants will complete them independently online through Google Forms, which will be sent to their email addresses immediately after each physiotherapy assessment.

#### 2.2.4. Follow-Up

Assessments will be conducted at four time points by the same blinded physiotherapist (PT1): before the intervention (A0), after the physiotherapy intervention (A1), and at three follow-ups, 3-, 6- and 12- months post-treatment (A2, A3, and A4, respectively).

The assessment appointment will be scheduled with them, and a reminder will be sent via SMS and email. If they are unable to attend, they will have access to a phone number and email address to contact, and another appointment will be scheduled with them.

#### 2.2.5. Intervention

Participants will be randomly assigned in a 1:1 ratio to one of two intervention groups using a computer-generated randomization sequence (Epidat 4.2, Xunta de Galicia, Spain). To ensure allocation concealment, the randomization sequence will be generated in advance by an independent researcher and stored in sealed, opaque, consecutively numbered envelopes. These envelopes will be securely kept and opened only by the designated physiotherapist (PT2) at the time of each participant’s enrollment. PT2 will not be involved in participant assessment or treatment to prevent potential allocation bias. Block randomization with variable block sizes will be used to maintain balance between groups throughout the study.

Participants will be allocated to either the active control group receiving multimodal physiotherapy (MP-G) or the experimental group receiving multimodal physiotherapy including radiofrequency (RMP-G). Both interventions will be individualized and administered by physiotherapists specializing in Pelvic and Women’s Health (PT3 and PT4), who will remain blinded to group allocation to ensure assessor masking. The approximate session duration will be 45 min per session, twice a week for 6 weeks, totaling 12 sessions. Both groups will receive therapeutic education, specific PFMT, and conservative and/or invasive treatment for MPS, with the addition of radiofrequency in the RMP-G. The details of each treatment strategy are explained below.

- Therapeutic education (MP-G and RMP-G): Learning about the anatomy, physiology, and pathophysiology of the abdominopelvic cavity, pelvic floor dysfunctions, and risk factors through anatomical slides and audiovisual resources. This also includes the concept of pain and its associated factors, as well as individual strategies to manage pain and improve and/or protect the pelvic floor.
- PFMT (MP-G and RMP-G): Active, specific PFM exercises, guided intravaginally with one or two contacts by the physiotherapist. These exercises involve different types of contractions aimed at improving proprioception, strength, endurance, and relaxation capacity. Participants will be encouraged to perform PFMT at home at least twice a week, as well as to include the knack maneuver during perceived increases in intraabdominal pressure.
- Conservative and Invasive Treatment of MPS (MP-G and RMP-G): After diagnosing MPS, active MTrPs will be treated through conservative techniques: dynamic and static compression of the MTrPs, local longitudinal massage of the muscle taut band, fascial massage of adjacent muscles, and local stretching of muscle fibers. If, after two sessions, the pain of the active MTrPs does not improve, dry needling will be used. Latent MTrPs will be treated with conservative techniques [39].
- Non-ablative radiofrequency (RMP-G): The treatment will begin in the third session, using capacitive superficial electrodes (Ø35 mm) (which penetrate less deeply into the tissue) and resistive superficial (Ø30 mm) and intravaginal (longitudinal, Ø14 mm) electrodes (for deeper tissues). A capacitive electrode protocol will be applied for 5–10 min using circular movements over the scar region (if present), the perineal body, and the labia majora with a bio-stimulation effect and a participant’s perception of heat of 0–2 on a 10-point scale for 2–3 min; and with a vascularization effect and a participant’s perception of heat of 6–7 on a 10-point scale for 2 min. This will be followed using a resistive external and intravaginal electrode, which promotes vascularization of the PFM, with a heat sensation of 6–7 on a 10-point scale for 15–20 min. The external resistive electrode will be applied in circular motions over the same areas as the external capacitive electrode, as well as the suprapubic region, while combining this with PFMT and conservative treatment for MPS. The intracavitary resistive electrode will perform a “U” movement along the vaginal fornix, pausing at areas with muscle taut bands and combining this with PFMT. The session will conclude with the application of a capacitive surface electrode for 5 min on the scar tissue and perineal body, with a subjective heat sensation of 0–2 on a 10-point scale. A radiofrequency device INDIBA^TM^ ACTIV 7 (Indiba S.A, Barcelona, Spain) will be used. This device operates at a fixed frequency of 448 kHz, with a maximum power of 250 VA in capacitive mode and a maximum power of 130 W in resistive mode. The intensity will be set based on the patient’s subjective perception, ranging from 0 to 2 for bio-stimulation effects, and 6 to 7 for a heating sensation to achieve a vascularizing effect.

To assess the safety of the interventions, in all treatment sessions, the physiotherapists performing the treatments (PT3 and PT4) will record any potential incidents, such as discomfort, increased pain, use of medication, need for additional medical consultations, postponement, or suspension of treatment due to possible adverse effects of the interventions.

#### 2.2.6. Statistical Data Analyses

A descriptive analysis of each collected variable will be conducted. For continuous variables, means, medians, standard deviations, and quartiles will be calculated, depending on whether the assumption of normality is met, as determined beforehand using the Shapiro–Wilk test. Qualitative variables will be described with absolute and relative frequencies in percentages.

For intragroup comparisons over time (A0–A4), if continuous variables follow a normal distribution, repeated-measures ANOVA will be used. If the data do not follow a normal distribution, the Friedman test will be used. For ordinal variables (FSFI, PFDI-20, PFIQ-7, Broome Scale), the Friedman test will be used for comparisons across more than two time points, or the Wilcoxon signed-rank test for comparisons between two time points, regardless of whether they follow a normal distribution. For dichotomous variables, the McNemar test will be applied for two time points, and the Cochran Q test will be used for multiple time points.

To determine the association between groups at each time point, a dichotomous independent variable and a quantitative dependent variable with a parametric distribution, the independent Student’s t-test will be used if the data follow a parametric distribution. If the assumption of normality is violated, the Mann–Whitney U test will be applied. For ordinal variables, the Mann–Whitney U test will be used to compare two independent groups, and the Kruskal–Wallis test will be applied for comparisons across more than two groups. For dichotomous variables, the Chi-square test will be used, and Fisher’s exact test will be applied if the expected frequency is less than 5. The effect will be assessed by the difference in means, and the precision will be measured using the 95% confidence interval.

The main analysis to compare the groups will be adjusted to the baseline pre-intervention values of the outcome variables. In all cases, a significant level of *p* < 0.05 will be used. The statistical software SPSS, version 29, IBM, will be employed.

### 2.3. Qualitative Study

A qualitative study will be conducted from a phenomenological paradigm to know the postpartum women’s experience with adherence to the advice and home exercises recommended by their physiotherapist. The consolidated criteria for reporting qualitative research (COREQ) will be followed [47].

#### 2.3.1. Research Teams and Reflexibility

The individual interviews and focus groups will be conducted by two physiotherapists specialized in Pelvic and Women’s Health, who are women, mothers, university professors, and hold a PhD. Interviewer 1 (PT1) will be familiar with the participants in advance, as she is the blinded evaluator of the RCT. This evaluator will interview the women who have already completed the follow-up in order to avoid conflicts related to the potential disclosure of the intervention group during the interviews. Interviewer 2 (PT2) will not know the participants in advance and will conduct interviews with women who are between the A2 and A4 follow-up assessments. Both evaluators have prior experience conducting individual interviews and focus groups within the framework of qualitative studies in women’s health.

#### 2.3.2. Qualitative Study Design

The theoretical framework and methodological orientation for the qualitative part of the RASDOP study is based on phenomenology. The main objective will be to understand the experience of postpartum women in adhering to a physiotherapy treatment aimed at improving their postpartum pelvic pain.

Women who have completed the 12 individual physiotherapy sessions will be included, regardless of the assigned treatment group of the RCT. Women will be divided into two groups: one consisting of women who are in the follow-up period (3 to 12 months after completing the intervention), or those who have already finished their participation in the study (> 12 months after the physiotherapy intervention). The recruitment will be consecutive, and the study will end when data saturation is reached. Data saturation will be deemed achieved when the information gathered aligns with that from earlier interviews.

All interviews will be conducted face-to-face and will take place in a classroom in the Nursing and Physiotherapy Building at the University of Alcalá, near the FPSM Research Group laboratory. The focus groups will consist of four to six women who do not know each other beforehand. In the interviews, only the participating postpartum women and one of the interviewers (PT1 or PT2) will be present. The interviewers will use a pre-designed question guide (Table 3), and the sessions will be audio-recorded with the prior consent of the participants.

#### 2.3.3. Analysis and Findings

The interviewers, along with three other physiotherapists, two specializing in Pelvic and Women’s Health (PT4 and PT5) and one expert in qualitative research (PT6), will transcribe the recorded interviews. The data will be coded to maintain the participants’ anonymity, based on whether the interview is individual or group-based, and according to the order of participation. The researchers who perform the transcriptions will reach a consensus on the identified themes and group them into categories. For transcription, coding, and categorization of the information extracted from the interviews, the software ATLAS.ti 25 (Scientific Software Development GMBH, Berlin, Germany) will be used.

## 3. Results

The RASDOP study aims to assess the effectiveness and safety of including non-ablative radiofrequency as part of a multimodal physiotherapy treatment for postpartum pelvic pain. Additionally, the study seeks to understand the experiences of these women regarding adherence to conservative treatment and home-based advice.

### 3.1. RASDOP Randomized Controlled Clinical Trial Expected Results

The multimodal physiotherapy intervention, based on therapeutic education, PFMT, treatment of MPS in the abdominal and/or PFM, and the use of radiofrequency in women with pelvic pain after childbirth, is expected to significantly improve pelvic floor symptoms in these women. Thus, the RASDOP study is proposed as a solution to improve symptoms related to pelvic pain, the potential occurrence of urinary incontinence, increased urinary frequency, or even the presence of involuntary gas leakage in the postpartum period.

A significant improvement is expected in sexual function, particularly in the domains of lubrication, orgasm, and pain, as assessed by the FSFI questionnaire. Regarding distress and the impact of other pelvic floor dysfunctions, such as lower urinary tract issues, pelvic organ prolapse, and anorectal dysfunctions, evaluated through the PFDI-20 and PFIQ-7 questionnaires, a decrease of more than a 23% in the scores is anticipated, indicating an improvement in symptoms and a reduced impact on quality of life [48].

An increase in the strength of the PFM is expected, as measured by vaginal dynamometry, due to the performance of PFMT and the adoption of healthy PFM protection habits. In contrast, an improvement in the relaxation capacity and elasticity of the PFM is anticipated, as assessed by transperineal ultrasound.

It is expected that the presence of MTrPs in the abdominal muscles and PFM will decrease or even be eliminated. In cases where MTrPs are still palpated, it is anticipated that they will be latent and not trigger painful symptoms in the women.

The Broome scale will be used to assess self-confidence and self-efficacy in performing PFMT, as well as changes in lifestyle behavior, which are expected to help sustain treatment benefits in the long term. It is anticipated that participants in both groups will achieve a score of over 66 points on the Broome scale during the post-treatment and follow-up evaluations, indicating a high perception of self-efficacy [46].

Although both intervention groups are expected to show significant improvements between the pre- and post-treatment assessments, and maintain their results at 3-, 6- and 12-month stages post-treatment, it is anticipated that these improvements will be greater in all variables in the RMP-G group, particularly in sexual function, pain reduction, and the lower presence of MTrPs.

No adverse effects are expected in either intervention group. The authors believe that interventions will not need to be postponed, nor will participants require analgesic medication.

### 3.2. RASDOP Qualitative Study Expected Results

The postpartum period is a vulnerable time during which women must balance their physical and mental recovery from pregnancy and childbirth with their new role and responsibilities as mothers. This qualitative study aims to delve into the experiences of postpartum women, exploring the facilitators and barriers they encounter in undertaking a conservative treatment to address their postpartum pain symptoms and adhering to healthcare recommendations. This will enable clinicians to propose interventions that are not only effective and safe but also tailored to the unique challenges faced by women during the postpartum period.

## 4. Discussion

To the authors’ knowledge, this is the first RCT with evaluator blinding to compare the effectiveness of a multimodal physiotherapy treatment versus a multimodal treatment that includes non-ablative radiofrequency in managing pelvic pain and sexual function from six weeks to six months postpartum. The significance of the RASDOP study lies in addressing the fact that nearly 50% of women develop symptoms of pelvic pain and/or dyspareunia following childbirth [12,13]. If left untreated, this pain can persist over time [49], negatively impacting sexual function, mental health [50], quality of life, and even the desire to have more children.

Previous studies have evaluated the efficacy of radiofrequency in pelvic pain immediately after childbirth [51,52]. These include two double-blind RCTs comparing the immediate use of radiofrequency versus placebo in women following vaginal delivery. Two consecutive sessions were conducted during the participants’ hospital stay, and perineal pain was assessed at rest, while sitting, and during walking. Bretelle et al. [51] reported improvements in walking pain and reduced analgesic consumption in the study group. In contrast, Sierenska et al. [52] found that radiofrequency improved pain and discomfort at rest, while sitting, and while walking but did not observe a reduction in the consumption of paracetamol or ibuprofen. In the acute phase, perineal trauma can be the primary source of pain, which may sometimes improve with medication or local cooling [53]. However, this pain can persist over time, potentially involving other structures, such as the PFM, as the primary source of discomfort. The RASDOP study proposes applying radiofrequency not only to scars but also to the PFM and abdominal muscles, aiming to improve the MTrPs in these muscles. Radiofrequency has only demonstrated short-term effects, and existing studies have heterogeneous protocols, small sample sizes, or lack a control group [28]. Therefore, more evidence is needed to support the use of radiofrequency for pelvic pain, as well as the additional financial cost associated with this technology. Additionally, radiofrequency is a passive treatment technique, while maintaining long-term benefits requires behavioral modifications and the adoption of active treatment strategies [54]. Therefore, the RASDOP study will include educational management and PFMT in both treatment groups.

Descriptive studies in women experiencing pelvic pain during the postpartum period report alterations in the PFM, including decreased strength and neuromuscular activation [44], reduced tone [44,55], increased tenderness, delayed voluntary activation of the PFM, and abdominal changes such as increased interrectus distance and greater distortion of the linea alba [55]. MPS is a highly prevalent clinical condition in musculoskeletal disorders and has also been shown to be present in various persistent pelvic pain syndromes, such as in women with endometriosis [56] or primary dysmenorrhea [57]. Moreover, the presence of MPS in the PFM has been diagnosed in up to 85% of women with pelvic floor dysfunctions. It has been associated with an increased sensation of pelvic pressure and heaviness in women with pelvic organ prolapse, as well as pain in the lower abdomen and difficulty emptying the bladder in women with urinary symptoms. [19]. Similarly, tenderness in the PFM is associated with dyspareunia and sexual dysfunction, with MPS acting as a perpetuating factor for pain during sexual intercourse [58]. Li et al. [29] also focused on treating MPS in the PFM during the postpartum period, comparing the use of radiofrequency with manual treatment of MTrPs. Both groups achieved pain reduction with no statistically significant differences between them. However, the group treated with radiofrequency experienced an increase in vaginal laxity and improved perceived comfort. Nonetheless, neither group showed improvements in the contractile capacity or neuromuscular activity of the PFM. This may have been due to the absence of specific PFMT in the intervention protocol. The RASDOP study proposes a multimodal physiotherapy treatment that includes PFMT alongside an educational strategy. The aim is to enhance PFM properties as both a protective and therapeutic factor for MPS, which will also contribute to improving sexual function [59]. This approach aligns with international recommendations for preventing pelvic floor dysfunctions during the postpartum period [30].

The classification of pelvic pain is controversial and varies depending on the source consulted. This study will include women with pelvic pain without infection or obvious local pathology, which, according to the International Association for the Study of Pain (IASP) classification, is considered primary pelvic pain syndrome [1]. Since the study will assess the presence of MPS in PFM and abdominal muscles, we will follow the classification provided by the European Association of Urology [2], based on IASP recommendations. Therefore, primary pelvic floor muscle pain syndrome and coccyx pain syndrome, both associated with musculoskeletal pain, will be included.

Among the strengths of the RASDOP study is its design as an RCT conducted by experienced physiotherapists specializing in clinical practice and research in Pelvic and Women’s Health. Participants will be referred by a gynecology service that has maintained a referral relationship for over 15 years, ensuring high-quality conservative treatment for women—a service currently not provided free of charge in Spain. The study’s rigorous design will allow for the evaluation of the efficacy and safety of incorporating radiofrequency technology, an increasingly utilized intervention in clinical settings but with limited supporting evidence to date. Another notable strength is the comprehensive outcome measures to be collected. These will include subjective aspects and quality-of-life impacts, as well as objective local physical changes in the pelvic floor. This will be the first study to assess changes in sexual function and other pelvic floor dysfunctions, such as urinary and/or anal incontinence and pelvic organ prolapse, as well as changes in PFM tone, strength, and morphology in postpartum women experiencing pain who undergo physiotherapy treatment that incorporates radiofrequency. Additionally, the follow-up period will span one year, whereas published studies in this field have only reported immediate post-intervention results [29,51,52].

As a limitation, considering the multifactorial etiology of postpartum pain, hormonal factors may predispose women to greater sexual dysfunction, such as genitourinary syndrome of lactation [60]. To address this, variables related to changes in breastfeeding and the return of menstruation will be collected, as these factors may influence the presence or absence of pelvic pain and sexual function. Additionally, the personal process of role transitions that women experience during this stage of life may play a significant role in sexual dysfunction, beyond purely physical factors [61]. Therefore, another limitation of this study is the absence of a prior neuropsychiatric evaluation. To mitigate this, participants will be asked whether they have previously attended or are currently attending a mental health service. For immediate postpartum perineal pain, the standard medication protocol consists of paracetamol or non-steroidal anti-inflammatory drugs as needed [62,63]. Since medication use will not be altered within the study, any intake by participants will be recorded at each assessment.

If the multimodal physiotherapy treatment that includes radiofrequency is proven to be effective and safe in managing postpartum pelvic pain and sexual function, this conservative therapeutic strategy could be widely implemented to support these women. Furthermore, the qualitative study will provide insights into the factors influencing adherence to healthy habits and home exercises among women experiencing postpartum pain. This knowledge will facilitate the development of treatment programs designed to ensure long-term effectiveness by promoting patient adherence and motivation.

## 5. Conclusions

The RASDOP study will determine whether the inclusion of radiofrequency in a multimodal physiotherapy treatment—based on PFMT, therapeutic education, and the management of MPS in the abdominal and PFM—enhances its effectiveness in relieving pain and improving sexual function in postpartum women. Additionally, it will explore the barriers and facilitators that influence postpartum women’s adherence to conservative treatment and home-based recommendations for alleviating pelvic pain.

## Figures and Tables

**Table 3:** Proposed interview guide for the RASDOP study.

Number	Questions
1.	What has been your experience participating in this physiotherapy program?
2.	What motivated you to participate in this physiotherapy program? What were your goals or objectives? What did you aim to achieve? Do you think you have achieved them?
3.	From your perspective, what aspects have made it difficult to achieve the goals or objectives of this program? How could we have supported you better?
4.	Was it easy or difficult for you to attend the treatment and evaluation sessions? Why? Have we made it easier for you in any way? Is there anything that could have made it even easier?
5.	Have you been able to apply the knowledge gained during the treatment or evaluation sessions? What facilitated this? What made it more difficult?
6.	Have you adopted or modified any habits that you believe have improved your pelvic health? What about other aspects of your general health? Which ones? Was it easy to adopt these habits? Why? Was it difficult? Why?
7.	Do you believe that attending pelvic floor physiotherapy has been important for your recovery? Why? Do you think you would have needed any other treatments, guidance, advice, or modifications to the program? Are there any treatments, guidance, or advice that you consider unnecessary?
8.	What were and are your expectations regarding the improvement in pelvic pain? Do you think you have done or are doing anything to help alleviate it? Do you feel that you need or would have needed anything else?
9.	Do you think there is anything more or different we could do to help women experiencing pelvic and/or perineal pain postpartum?

The first question from each numbered set will be asked. Follow-up questions will be posed as needed based on the flow of the interview.

## Data Availability

The original contributions presented in this study are included in the article; further inquiries can be directed to the corresponding author.
